# High Energy Density and Temperature Stability in PVDF/PMMA *via In Situ* Polymerization Blending

**DOI:** 10.3389/fchem.2022.902487

**Published:** 2022-05-19

**Authors:** Yongbin Liu, Zhengwei Liu, Jinghui Gao, Ming Wu, Xiaojie Lou, Yanhua Hu, Yong Li, Lisheng Zhong

**Affiliations:** ^1^ State Key Laboratory of Electrical Insulation and Power Equipment, Xi’an Jiaotong University, Xi’an, China; ^2^ Frontier Institute of Science and Technology, State Key Laboratory for Mechanical Behavior of Materials, Xi’an Jiaotong University, Xi’an, China; ^3^ Department of Chemical Engineering, Ordos Institute of Technology, Ordos, China; ^4^ Inner Mongolia Key Laboratory of Ferroelectric-Related New Energy Materials and Devices, School of Materials and Metallurgy, Inner Mongolia University of Science and Technology, Baotou, China

**Keywords:** energy density, energy storage dielectrics, temperature stability, *in situ* polymerization, polyvinylidene fluoride

## Abstract

Dielectrics with improved energy density have long-standing demand for miniature and lightweight energy storage capacitors for electrical and electronic systems. Recently, polyvinylidene fluoride (PVDF)-based ferroelectric polymers have shown attractive energy storage performance, such as high dielectric permittivity and high breakdown strength, and are regarded as one of the most promising candidates. However, the non-negligible energy loss and inferior temperature stability of PVDF-based polymers deteriorated the energy storage performance or even the thermal runaway, which could be ascribed to vulnerable amorphous regions at elevated temperatures. Herein, a new strategy was proposed to achieve high energy density and high temperature stability simultaneously of PVDF/PMMA blends by *in situ* polymerization. The rigidity of the amorphous region was ideally strengthened by *in situ* polymerization of methyl methacrylate (MMA) monomers in a PVDF matrix to obtain PVDF/PMMA blends. The atomic force microscopic study of the microstructure of etched films showed the ultra-homogenous distribution of PMMA with high glass transition temperature in the PVDF matrix. Consequently, the temperature variation was remarkably decreased, while the high polarization response was maintained. Accordingly, the high energy density of ∼8 J/cm^3^ with ∼80% efficiency was achieved between 30 and 90 °C in PVDF/PMMA films with 39–62% PMMA content, outperforming most of the dielectric polymers. Our work could provide a general solution to substantially optimize the temperature stability of dielectric polymers for energy storage applications and other associated functions.

## Introduction

Energy storage devices with dielectric material play a critical role in electronics and electrical power systems such as hybrid vehicles, renewable energy systems, and electromagnetic systems (Tan, 2020; Wu et al., 2022). The storage of electrostatic energy depends on electric-field–induced polarization of dielectric materials, requiring high dielectric permittivity, high breakdown strength, and low loss. Polymers are one of the most widely used dielectric materials due to their advantages of high breakdown strength and scalability. Nowadays, the demand for higher energy and energy storage capacitors with higher power is rapidly growing. Taking hybrid vehicles as an example, the energy storage capacitors are required to be lightweight and should be able to withstand high temperatures of the nearby heat source like engines (Li et al., 2021; Li et al., 2016). This makes it highly challenging to prepare next generation dielectric polymers with high energy density and high temperature stability.

The energy density (*u*_e_) stored in dielectric materials can be presented by the following equation:

$$u_{e}=\int EdD,$$

where *E* is the electric field, and *D* is the electrical displacement (Chu et al., 2006). It is evident that energy density mainly depends on the breakdown strength (*E*_b_) and polarization response (*P*) of dielectric materials. At present, biaxially oriented polypropylene (PP) is the most widely used dielectric material for energy storage, owing to its high breakdown strength, low dielectric loss, and ease of manufacturing (Zheng et al., 2018). However, the shortcomings of polypropylene are its inherent low permittivity (*ε*_r_ ≈ 2.2) and consequently low energy density (∼2 J/cm^3^). Recently, poly (vinylidene fluoride) (PVDF) and its copolymers have gained a lot of attention, owing to their high permittivity (*ε*_r_ ≈ 10–50), which enables them to achieve a high energy density (4−30 J/cm^3^) (Prateek et al., 2016; Baer and Zhu, 2017; Zha et al., 2021). However, at elevated temperature, the working voltage rate for PVDF-based dielectrics must be reduced without extra cooling devices due to their poor temperature stability (Li et al., 2015; Li et al., 2021).

Such a dilemma arises due to the contrasting features between the high energy density and robust temperature stability required for the mobility of molecular chains. To achieve high energy density, the mobility of activated molecular chains favors easy switching of polarization under external electric fields and consequently high polarization response. On the other hand, to maintain robust temperature stability, freezing of molecular motion is preferred to prevent an upsurge of current leakage and free volume (Rajib et al., 2015; Zhou et al., 2018; Zhou et al., 2020; Liu et al., 2022). Therefore, the energy storage performance of PVDF-based dielectrics is usually exciting at room temperature. However, the elevation of working temperature up to 70°C, results in a drop of energy density by 22–80% (Khanchaitit et al., 2013; Rajib et al., 2015; Li et al., 2016; Liu et al., 2017). Research works on enhancing temperature stability of polymer dielectric focus on the rigidity of chains through either chemical means or physical approach involving the spatial confinement effect. For example, Li et al. crosslinked poly (chlorotrifluoroethylene-co-vinylidene fluoride), which showed improvement in the energy density and efficiency from 0.57 J/cm^3^ and 37.9% to 4.33 J/cm^3^ and 70% at 150°C, respectively (Li et al., 2020). A variety of polymers with aromatic groups or fused-ring heterocyclic configurations on backbones, such as polyetherimide (PEI), polyether ether ketone (PEEK), and polytetrafluoroethylene (PTFE), have been investigated at higher temperatures (above 150°C) (Li et al., 2019; Zhang et al., 2021; Wu et al., 2022). The chemical modifications of polymer chains can significantly reinforce thermal stability but at the cost of polarization response. Using the physical approach, Zhu et al. laminated PVDF with polycarbonate (PC), thus enabling the working of layered materials at 170°C (Yin et al., 2019). In our previous work, blending of PVDF with high glass transition temperature (*T*_g_) polymethyl methacrylate (PMMA) introduced a spatial confinement effect on the structure upon heating, which brought about the stabilization of energy density around 7.8−9.8 J/cm^3^ between 30 and 70°C (Liu et al., 2019). However, the confinement provided by van der Waals forces of physical modification is inferior to that achieved through chemical approaches. Consequently, the realization of high energy density and robust temperature stability in polymer dielectrics remains a big challenge.

In this work, an *in situ* polymerization approach of PMMA in the PVDF matrix was proposed to achieve ultra-homogeneous distribution and thereby enhance its confinement effect on temperature stability. Blending by *in situ* polymerization was realized through the polymerization of MMA in the PVDF solution and confirmed by nuclear magnetic resonance spectroscopy and thermogravimetric analysis. It was evident that the enhanced homogeneous dispersion significantly improved the structural stability upon heating and thus improved the energy storage performance at elevated temperature. Our finding provides a general approach for developing high-performance polymer blends to achieve high energy density and robust temperature stability simultaneously.

## Experimental

### Materials

PVDF powder was purchased from Alfa Aesar, whereas MMA monomers were purchased from TCI. Azodiisobutyronitrile (AIBN), methanol, and *N*,*N*-dimethylformamide (DMF) were purchased from Aladdin, and acetic acid was purchased from Macklin.

### Sample Preparation

PVDF/PMMA blends were prepared by *in situ* polymerization. First, PVDF powder was dissolved in DMF in a 10% weight ratio. MMA monomer was added into the solution, according to its weight ratio in the blend (20 wt% to 80 wt%, 10 wt% per step, and 100%). After the solution was stirred to homogenize, it was cooled down to about -70°C and protected from light, followed by the addition of AIBN (1% of MMA). Second, the solution was polymerized at 75°C for 2 h under the nitrogen atmosphere. Finally, the samples were obtained as sediments from methanol and dried (Hussein et al., 2016; Hussein et al., 2018). PVDF/PMMA films (20 mm × 20 mm) were prepared by solution casting, followed by heat treatment at 200°C for 10 min, and then quenched with ice water.

### Characterization

The characteristic groups of PMMA were measured by nuclear magnetic resonance to confirm the successful polymerization of PMMA after the fabrication process. The successful polymerization of PMMA was verified using the 1H spectrum of nuclear magnetic resonance (NMR, JEOL 400 MHz). Atomic force microscopy (AFM, Bruker Icon) was employed to study the morphology of polymer films. Prior to observation, the blend films were pre-treated (etched) with glacial acetic acid for 1 h to remove the PMMA component, which helped to determine the distribution of PVDF and PMMA. The thermodynamic properties of the specimens were measured by thermogravimetric analysis (TGA, METTLER TOLEDO) and differential scanning calorimetry (DSC, Discover DSC250) at a heating rate of 10 °C/min. The crystalline structures of the specimens were analyzed by Fourier transform infrared spectrometry (FTIR, NICOLET6700) and X-ray diffractometry (XRD, Bruker Advance D8). To evaluate the variation of the structure with temperature rise, the *in situ* heating small-angle X-ray scattering (SAXS, Anton-Paar) measurement was conducted on the specimens with two-slit–collimated Cu–Kα radiation (*λ* = 1.54 Å). Dielectric permittivity and loss tangent were determined at 1 kHz using an LCR impedance analyzer (HIOKI IM3536), with circular gold electrode diameter = 10 mm. The electrical displacement (*D*)–electric field (*E*) hysteresis loops were obtained using a ferroelectric workstation (PolyK Technologies) with a 1,000 Hz unipolar triangular signal to determine the energy density and its associated efficiency with temperature changes, with circular gold electrode diameter = 3 mm.

## Results and Discussion

### Specimen Fabrication

**FIGURE 1:**
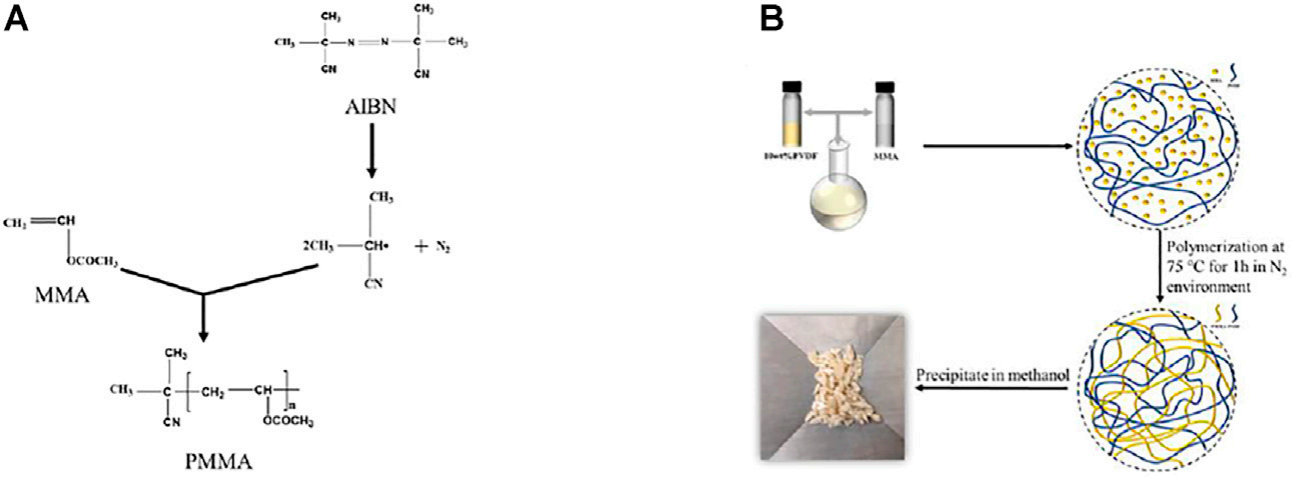
Schematic representation of the preparation process of PVDF/PMMA blends. **(A)** Synthesis process of PMMA and **(B)** the preparation process of PVDF/PMMA blends.

The *in situ* polymerization process of PMMA in the PVDF solution is depicted in Figure 1. The polymerization of MMA was initiated by AIBN at 75°C, and sufficient dispersion of PMMA was formed in the PVDF matrix. To confirm the successful polymerization of PMMA in the PVDF matrix, the PVDF/PMMA solutions are estimated by nuclear magnetic resonance which indicates the existence of characteristic -O-CH_3_ groups of PMMA, as shown in Supplementary Figure S1 (Mccord et al., 1994). Considering the possible variations during polymerization, the final weight ratios of the two phases in the blends were measured by TGA.

**FIGURE 2:**
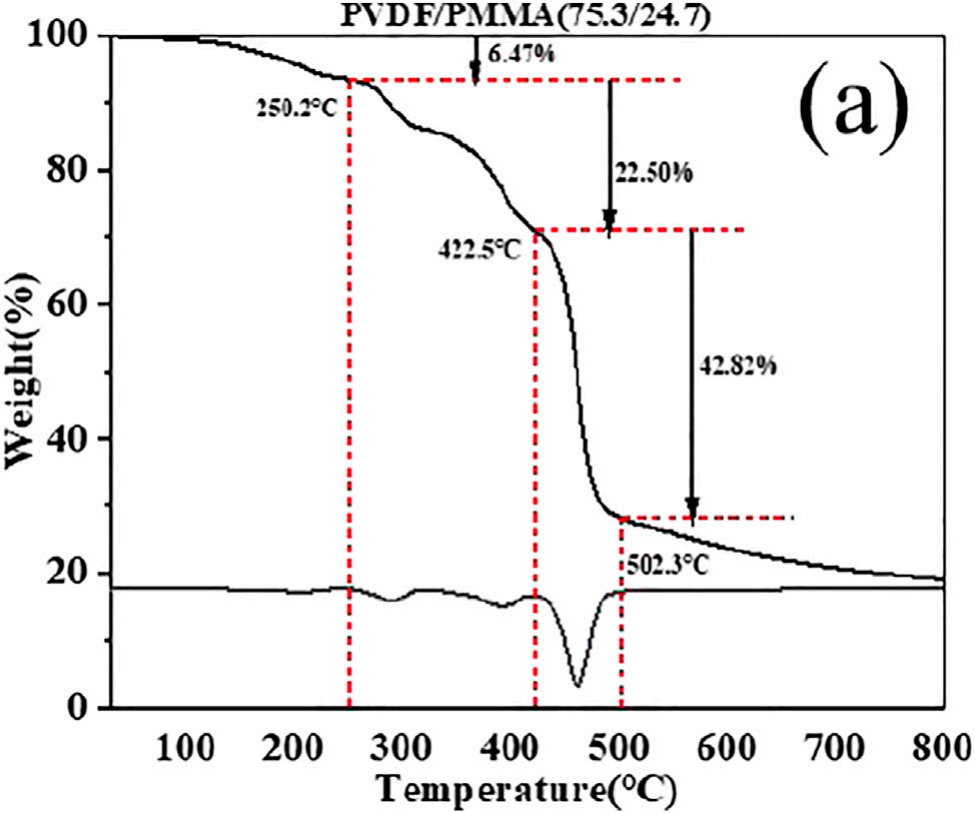
TG and DTG curves of the PVDF/PMMA blend (75.3/24.7)

As shown in Figure 2, PVDF/PMMA with a designed ratio of 70/30 was chosen as an example to determine the final weight ratio. PVDF/PPMMA blends underwent the four stages of thermal decomposition, which corresponded sequentially to the volatilization of residual DMF molecules, end-initiated scission of the unstable end groups of PMMA chains, random scission of PMMA chains, and decomposition of PVDF (Hirata et al., 1985; Botelho et al., 2008). Thereafter, PMMA lost 100% of its weight, whereas PVDF lost 62.5% (Nguyen, 1985). Accordingly, the proportion of PMMA (M(PMMA)) in the blends could be calculated by the following equation:

$$M\left(PMMA\right)=\frac{TG\left(PMMA\right)}{TG\left(PMMA\right)+\frac{TG\left(PVDF\right)}{62.4\%}},$$

where TG (PMMA) and TG (PVDF) are mass fractions of PMMA and PVDF, respectively. Thus, the weight ratio of PMMA was found to be 24.7%, slightly lower than the designed value of 30%. The TGA results of other samples are shown in Supplementary Figure S4, wherein the realized ratios of the blends were 0, 6.0, 19.5, 24.7, 39.0, 56.8, 62.1, and 74.3%. The PMMA ratio was evidently lower than its designed value, which could be ascribed to dilution of MMA by the PVDF solution.

### Crystallinity of the Polymer Blend

**FIGURE 3:**
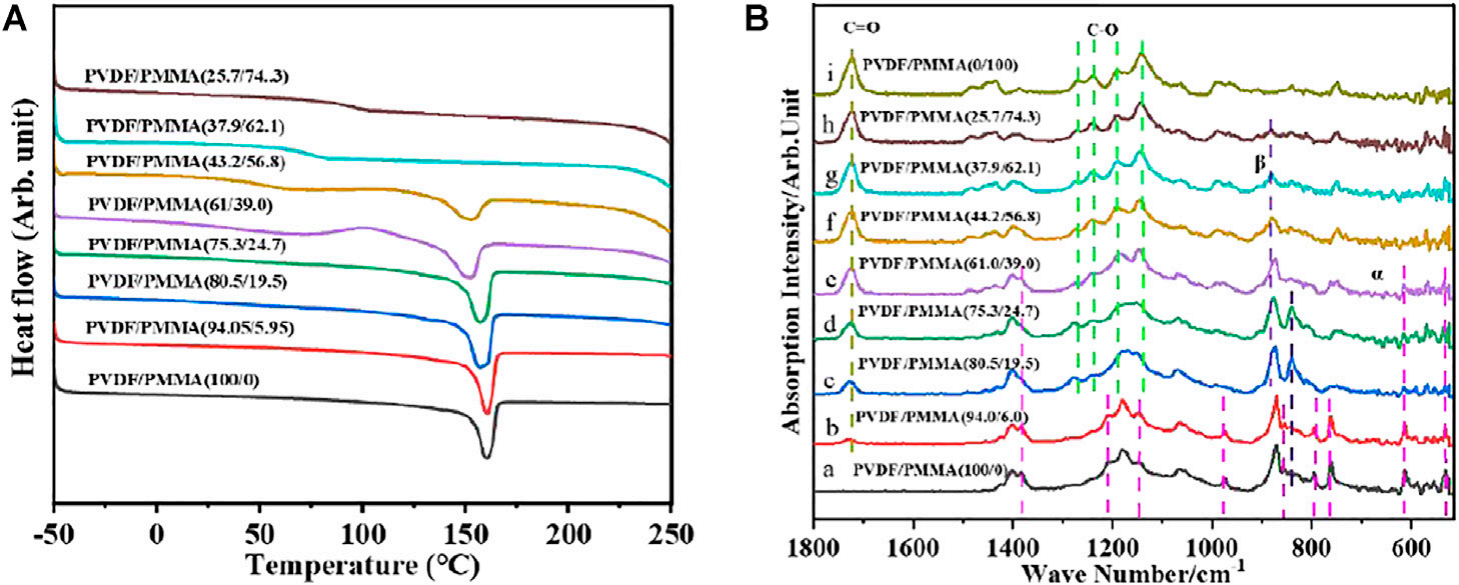
**(A)** DSC curves and **(B)** FTIR spectra of PVDF/PMMA specimens.

DSC and FTIR analyses were conducted to investigate the influence of *in situ* polymerization on the microstructure and crystallinity of the blend. The DSC profiles in Figure 3A indicate that the semi-crystalline feature of the blends gradually turned amorphous with the increase in the PMMA content. The crystallinity, derived from the melting enthalpy in the endothermic heat flow curve, decreased from 51% for neat PVDF to 4% for PVDF/PMMA (44/56), and the peak for melting enthalpy disappeared when the PMMA content was more than 56.8%. These results of crystallinity were further corroborated by FTIR spectral analysis, as displayed in Figure 3B. The characteristic absorption peaks for the *α*-phase of PVDF around 532, 614, 763, 795, 854, 975, 1,149, 1,209, and 1,383 cm^−1^ blurred when the PMMA content was above 39%, and those corresponding to the *β*-phase of PVDF around 840 and 881 cm^−1^ blurred when the PMMA content was above 74% (Cai et al., 2017). Therefore, the phase structure of PVDF turns from the α phase to the β phase gradually with PMMA addition, which is also evidenced by XRD results, as shown in Supplementary Figure S3. Also, both DSC and FTIR results suggested that the PVDF/PMMA blends experienced a drop in crystallinity and turned amorphous when the PMMA contents reached 56–74%. Furthermore, the melting temperature (*T*_m_) of the blends on the DSC profiles decreases with PMMA addition from 160.9°C for pristine PVDF to 151.8°C for PVDF/PMMA (43.2/56.8), suggesting the suppression of PVDF crystallization and imperfection of the PVDF crystalline structure. The amorphous regions were characterized by glass transition temperature, which manifests itself as a step in DSC profiles. Glass transition temperatures of PVDF and PMMA were about −40 and 105°C, respectively. In the DSC profiles of the blends, they merged into one step, which revealed exceedingly homogenous distribution of PVDF and PMMA in the amorphous regions due to *in situ* polymerization.

### Morphological Characterization

**FIGURE 4:**
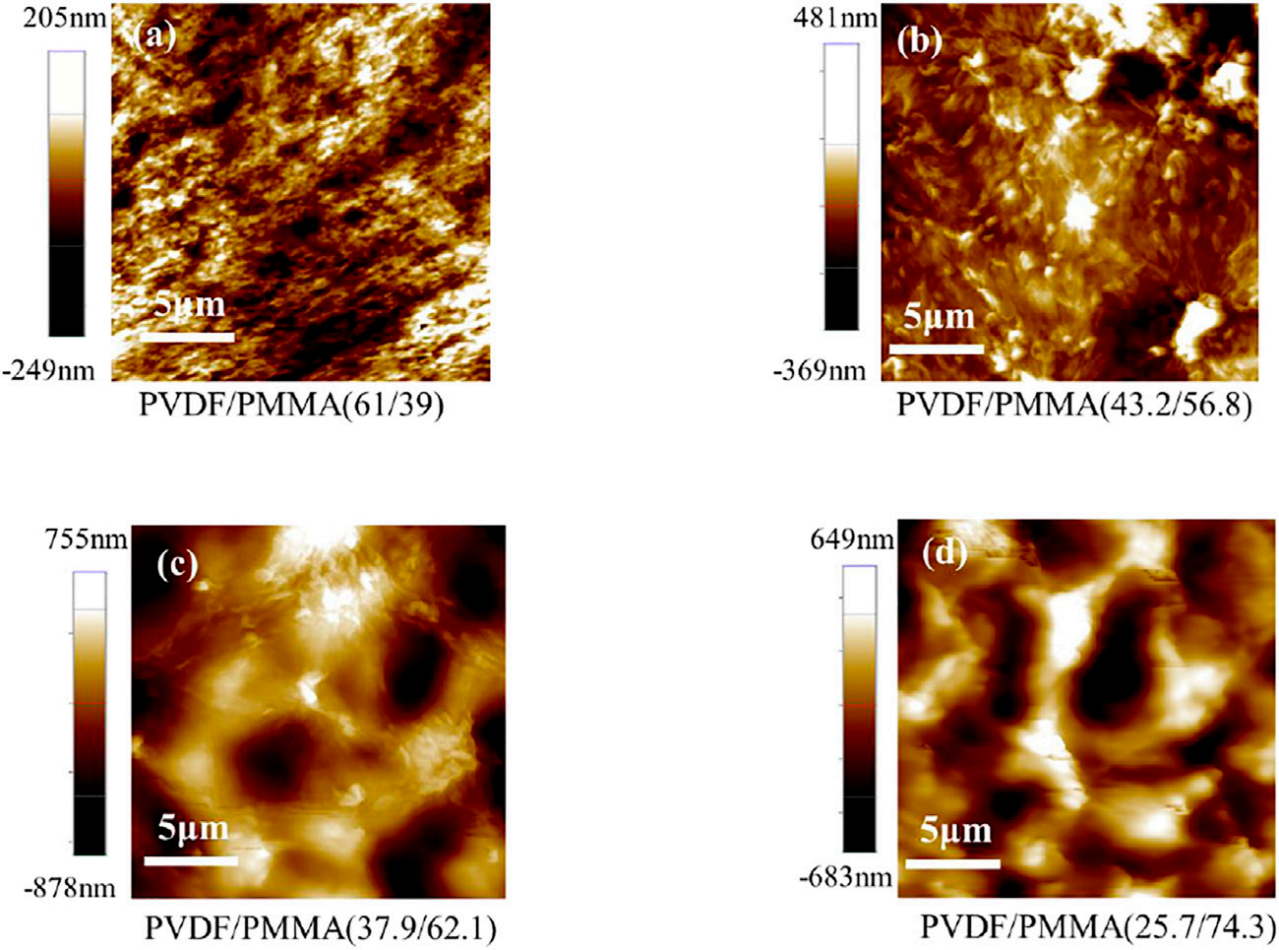
AFM height images of the blends with PMMA contents of **(A)** 39%, **(B)** 56.8%, **(C)** 62.1 and **(D)** 74.3% after etching.

AFM height profiles further helped to evaluate the distribution of PVDF and PMMA in the blends. Prior to AFM analysis, the blend films were pre-treated (etched) with glacial acetic acid for 1 h to remove the PMMA component. This procedure specifically dissolved PMMA and thus helped to determine the distribution of PVDF and PMMA. The bright yellow region in the AFM image indicated PVDF, and the dark yellow region indicated the cavity caused by the elimination of PMMA, as shown in Figure 4. The morphology revealed that the distribution of PVDF and PMMA was ultra-fine (∼μm) across the entire range of ratio, illustrating highly homogenous dispersion caused by *in situ* polymerization (Kang et al., 2009; Dong et al., 2019).

**FIGURE 5:**
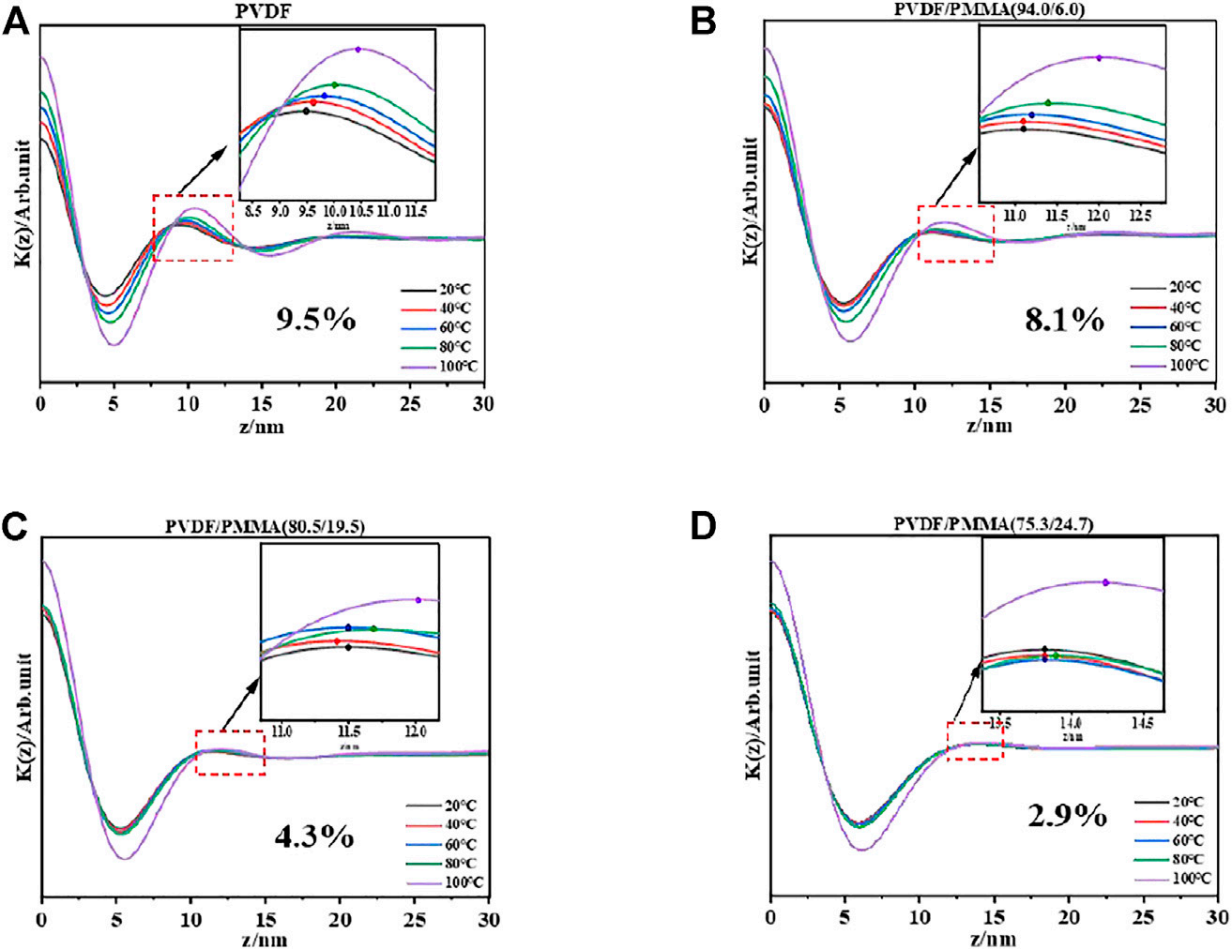
*K*(*z*) values from SAXS of the blends with PMMA contents of **(A)** 0%, **(B)** 6%, **(C)**19.5, and **(D)** 24.7%.

Homogenous dispersion could gently stabilize the structure upon heating by its adequate molecular contact (Wunderlich, 2003; Wei et al., 2015; Huang et al., 2018). To evaluate the confinement effect of *in situ* polymerization, the long period of PVDF/PMMA was assessed through one-dimensional correlation function (*K*(*z*)) of SAXS (Hu and Tashiro, 2016). The long period refers to the thickness of each lamella and amorphous layer in the crystalline stacks. Since the lamellar volume remains stable on heating below melting temperature, the variation over the long period mainly comes from the amorphous regions. The SAXS spectra of the PVDF/PMMA blends are shown in Supplementary Figure S5, in which the scattering peak of crystalline stacks diminished when the PMMA content reached 39%. Therefore, the one-dimensional fitting was only performed for the blends exhibiting an obvious scattering peak; the *K*(*z*) values of PVDF/PMMA with PMMA contents of 0%, 6%, 19.5, and 24.7% in the temperature range of 20–100°C are shown in Figure 5. The percentage of expansion for a long period was calculated by comparing its variations and is denoted in the figures.

Upon heating, the expansion of long period decreased from 9.5% for PVDF to 2.9% for PVDF/PMMA (75/25), demonstrating the enhanced rigidity of amorphous regions due to *in situ* polymerization (Fatnassi et al., 2010; Gumyusenge et al., 2018).

### Dielectric Responses

**FIGURE 6:**
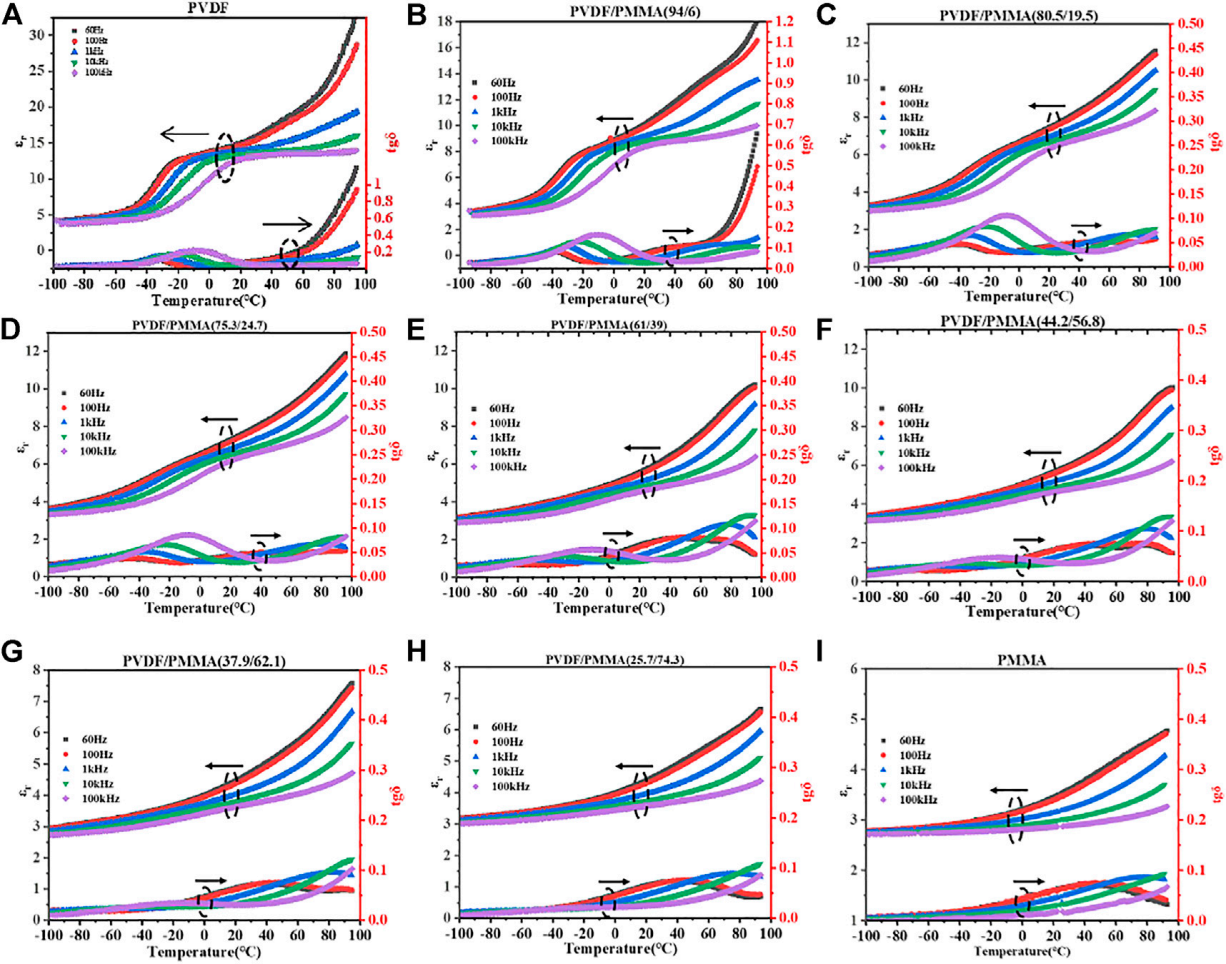
Temperature dependence of dielectric permittivity for PVDF/PMMA with PMMA contents of **(A)** 0%, **(B)** 6%, **(C)** 19.5%, **(D)** 24.7%, **(E)** 39%, **(F)** 56.8%, **(G)** 62.1%, **(H)** 74.3%, and **(I)** 100%.

Figure 6 shows variations in dielectric permittivity (*ε*_r_) and dielectric loss (tg*δ*) with temperature ranging from -100 to 90°C at different frequencies (60 Hz, 100 Hz, 1 kHz, 10 kHz, and 100 kHz). Although a decrease in permittivity on the addition of PMMA was inevitable, the dielectric loss at high temperature was significantly reduced. For pristine PVDF and PVDF/PMMA (94/6), dielectric loss increased rapidly when heated to above 60°C. With further addition of PMMA, the dielectric loss of the blends remained stable and low at elevated temperature. Such reduction certified the increase in rigidity by *in situ* polymerization of PMMA and was essential for the stabilization of energy storage performance upon heating.

### Energy Storage Performance

**FIGURE 7:**
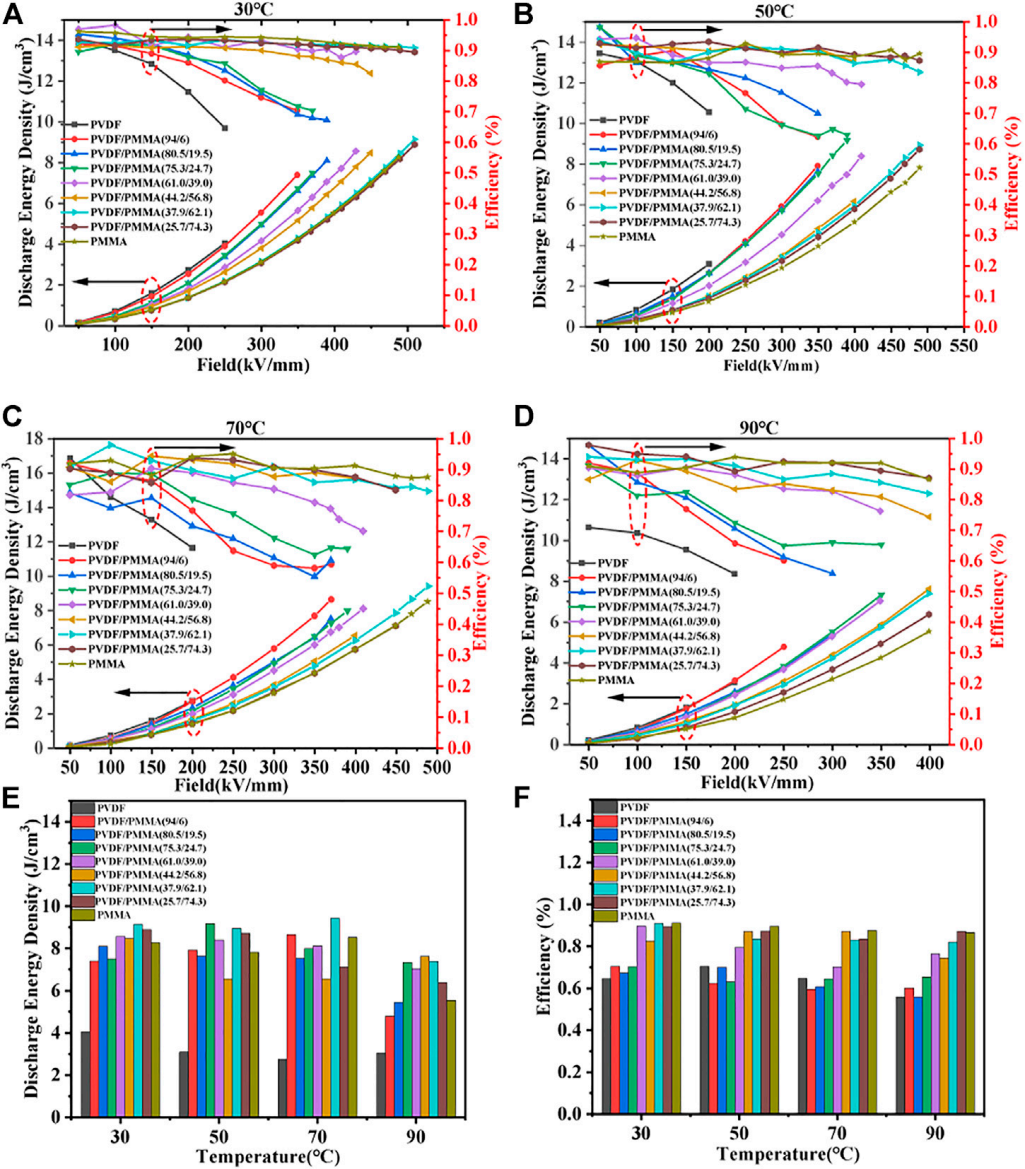
Energy densities and efficiencies of PVDF/PMMA blends at **(A)** 30°C, **(B)** 50°C, **(C)** 70°C, and **(D)** 90°C and the comparison of variations of **(E)** maximum energy density and **(F)** efficiency with temperature.

The energy storage performance of the blends from 30 to 90°C is shown in Figures 7A–D, whereas variations in maximum energy density and efficiency with temperature are compared in Figures 7E,F. With an increase in the PMMA content, in the same electric field, energy density decreased slightly due to decrease in polarization. However, in order to enhance the temperature stability of energy storage, it is more important to maintain stable breakdown strength and energy loss at elevated temperature. The blends with light PMMA (0–24%) showed an intense reduction in efficiency with an increase in the electric field and temperature, which could serve the energy dissipation and deteriorate the energy storage performance. As anticipated, efficiency remained stable with further increase in the PMMA content at elevated temperature and electric field. Consequently, the energy density of the blends with the PMMA ratio of 39–62% sustained around 8 J/cm^3^, and the efficiency of around 70–83% could be sustained within the testing temperature range.

It is noteworthy that the energy storage performance at elevated temperature was outstanding, compared with other dielectric polymers, as shown in Table 1. This verified the validity of our proposed *in situ* polymerization strategy for the blends.

**TABLE 1:** Comparison of energy densities of the blend prepared in this study with other dielectric polymers between 70 and 90°C.

Number	Polymer	*T* (°C)	*E* (kV/mm)	*U*_e_ (J/cm^3^)	*η*(%)	Reference
1	PVDF/PMMA(61/39)	70	410	8.1	0.7	This work
2	PVDF/PMMA(44.2/56.8)	70	400	6	0.83	This work
		90	400	7.6	0.74	This work
3	PVDF/PMMA(37.9/62.1)	70	490	9.4	0.83	This work
		90	400	7.4	0.82	This work
4	ABS	90	400	∼4.3	∼0.83	Wen et al. (2020)
5	PS	90	300	2.6	0.96	Wen et al. (2020)
6	P (TFE-HTP)	90	550	6.5	0.88	Zhang et al. (2017)
7	BOPP	70	400	2.1	0.9	Zhang et al. (2017)
8	NBT@AO/PVDF	80	400	7.46	∼0.5	Long et al. (2022)
9	PMBMP	85	400	∼3.7	∼0.8	Wang et al. (2020)

## Conclusion

In conclusion, an *in situ* polymerization blending strategy for the PVDF/PMMA blends was proposed with a view to improve the temperature stability of energy storage performance and unveil its influence on the structure and dielectric properties upon heating. Through *in situ* polymerization of high-*T*_g_ PMMA in the PVDF matrix, the blends displayed the ultra-homogenous distribution of the two components, leading to a robust amorphous structure upon heating, as characterized by SAXS. Accordingly, the energy density and efficiency were remarkably enhanced between 30 and 90°C. At elevated temperature, the energy density and efficiency of the blends with 39–62% PMMA were ∼8 J/cm^3^ and around 70–83%, respectively, which outperformed most relevant dielectric polymers at an elevated temperature. This work provides a general approach to synthesizing polar polymers with high energy density and robust temperature stability.

## Data Availability

The original contributions presented in the study are included in the article/Supplementary Material, further inquiries can be directed to the corresponding authors.
