# Supplementary material for: High Energy Density and Temperature Stability in PVDF/PMMA via In Situ Polymerization Blending
Source: Front Chem. 2022 May 19;10:902487. doi: 10.3389/fchem.2022.902487 (PMC9161359; doi:10.3389/fchem.2022.902487)
Supplement: Supplementary file 1 [file DataSheet1.docx]

Supplementary materials of High Energy Density and Temperature Stability in PVDF Blends via MMA In-situ Polymerization

# Nuclear Magnetic Resonance results

The PVDF/PMMA solution is estimated by nuclear magnetic resonance (NMR) to verify the successful polymerization of PMMA in the PVDF matrix. As shown in figure S1, the 1H spectrum of PVDF/PMMA (25.7/74.3) demonstrates the existence of characteristic -O-CH3 groups of PMMA, suggesting the formation of PMMA after polymerization.

**
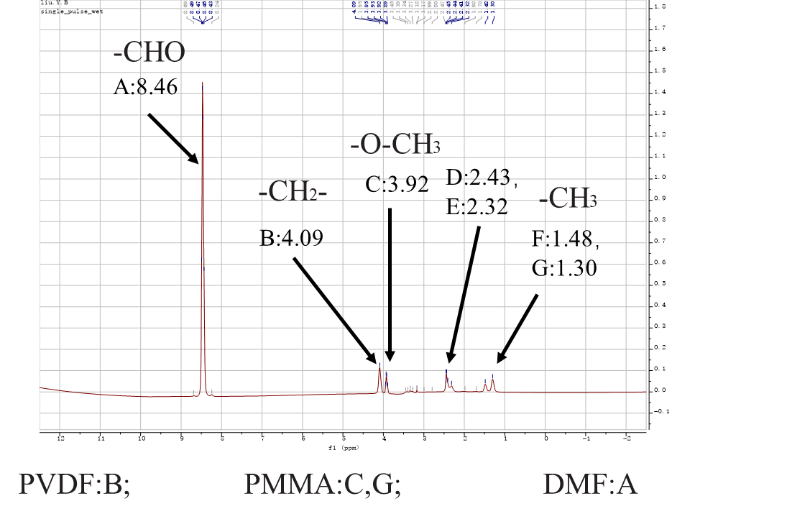
**

**Figure S1.** NMR 1H Spectrum of PVDF/PMMA (61/39)

# Surface Morphology

The surface morphology of PVDF/PMMA blends is observed by scanning electron microscopy (SEM) as shown in Figure S2. The crystalline structure can be observed when PMMA content is below 25% and then becomes blurred with a higher PMMA ratio. All the specimens exhibit smooth and homogenous surface morphology, indicating no obvious phase separation.

| **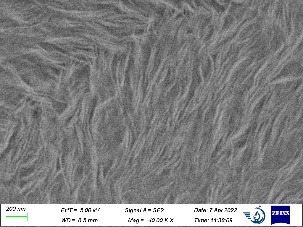** | **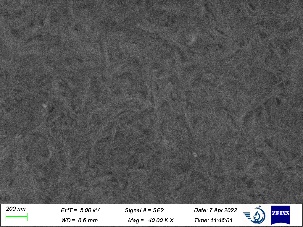** | **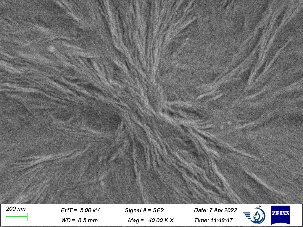** | **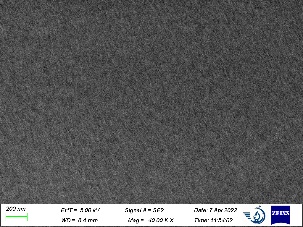** |
| --- | --- | --- | --- |
| a. Pristine PVDF | b. PVDF/PMMA(94/6) | c. PVDF/PMMA(80.5/19.5) | d. PVDF/PMMA(75/25) |
| 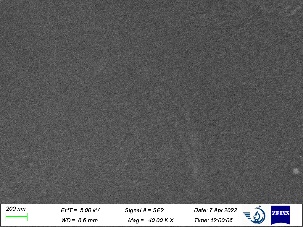 | 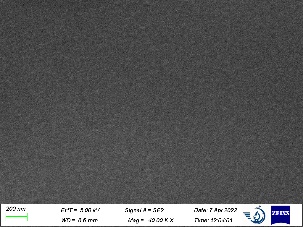 | 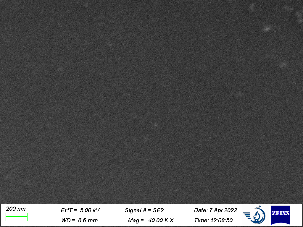 | 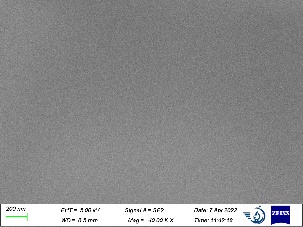 |
| e. PVDF/PMMA(61/39) | f. PVDF/PMMA(43.2/56.8) | g. PVDF/PMMA(37.9/62.1) | h. PMMA |

**Figure S2.** SEM images of surface morphology

# X-Ray Diffraction results

The X-Ray Diffraction profiles of PVDF/PMMA with varied PMMA contents are shown in Figure S3. The crystallinity decreases with PMMA addition and declines to around 0 when the PMMA ratio is above 40%, which is consistent with DSC results. For pristine PVDF and PVDF/PMMA(94/6), the XRD curves exhibit a characteristic α phase feature that diffraction peaks locate at 2*θ*=17.7°, 18.4°, 19.9°, and 26.7°. And the peaks locate at 2*θ*=20.0° and 20.4° for the blends with 19.5%~39% PMMA indicate a β phase feature. Therefore, the phase structure of PVDF turns from α phase to β phase gradually with PMMA addition. Such a change is also evidenced by FTIR results shown in Figure 3.

**Figure S3.** (a) XRD profiles of PVDF/PMMA and (b) the corresponding calculated crystallinity

# Thermogravimetric Analysis results

The PVDF/PMMA blends are estimated by Thermogravimetric Analysis (TGA) to calculate the final weight ratio of PMMA in production. As shown in figure S4, the final weight ratio of PMMA in production is 0%, 6.0%, 19.5%, 24.7%, 39.0%, 56.8%, 62.1% and 74.3%, respectively.

| **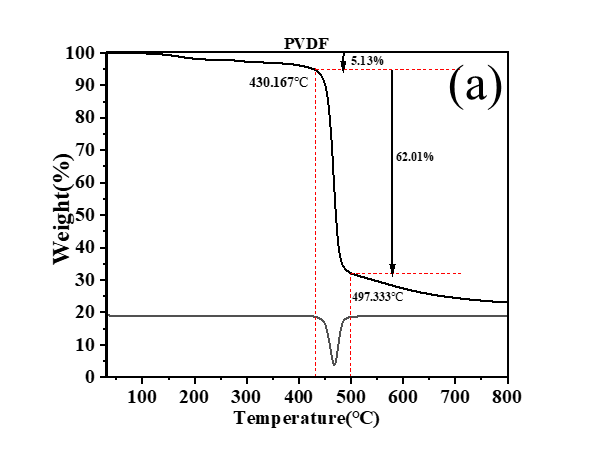** | **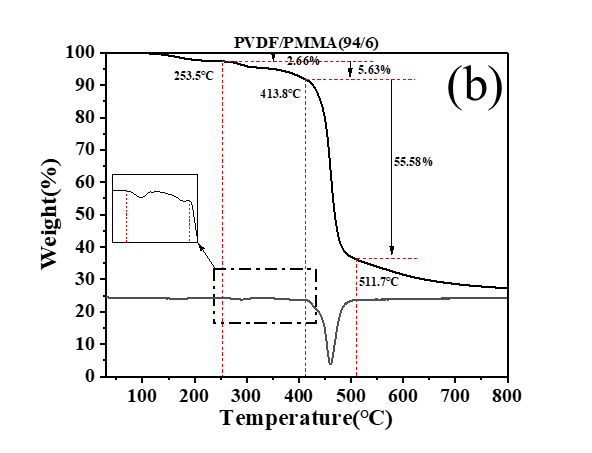** | **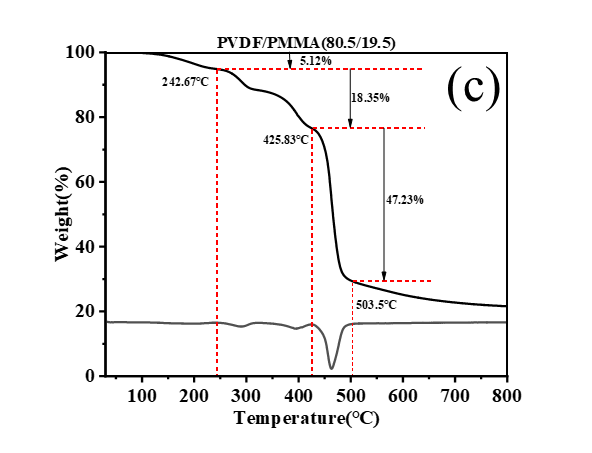** |
| --- | --- | --- |
| **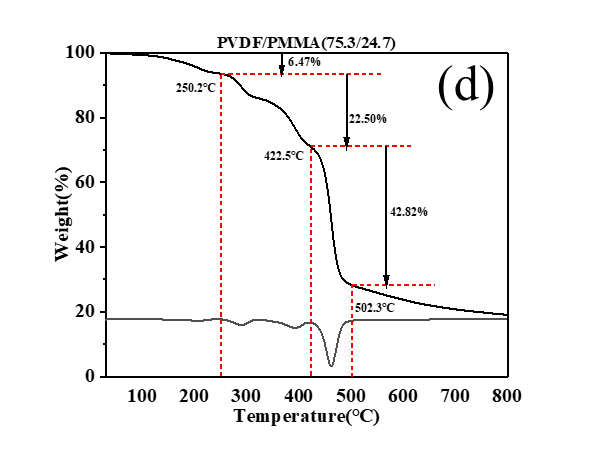** | **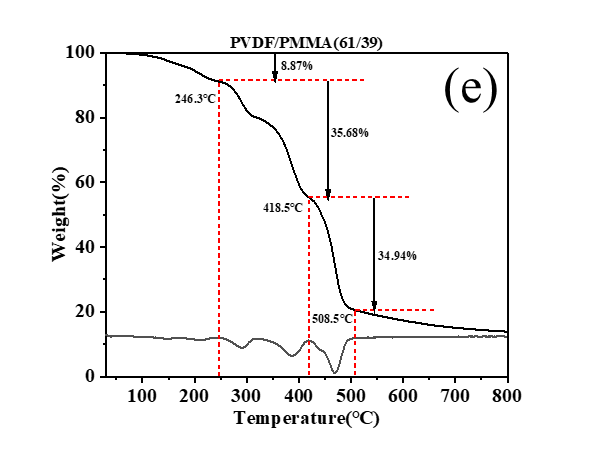** | **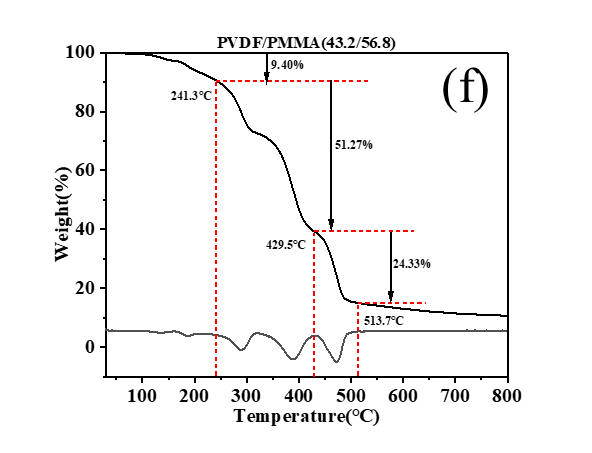** |
| **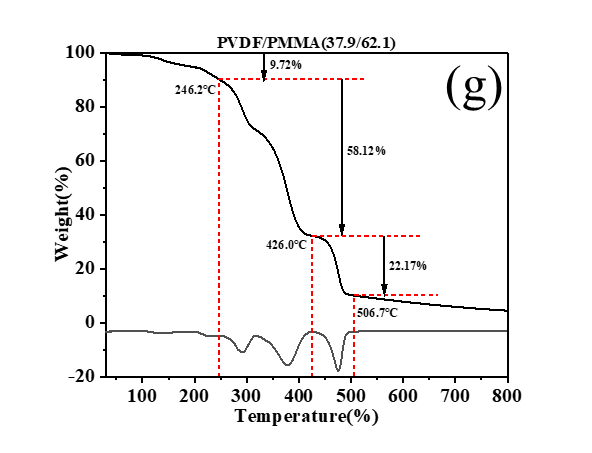** | **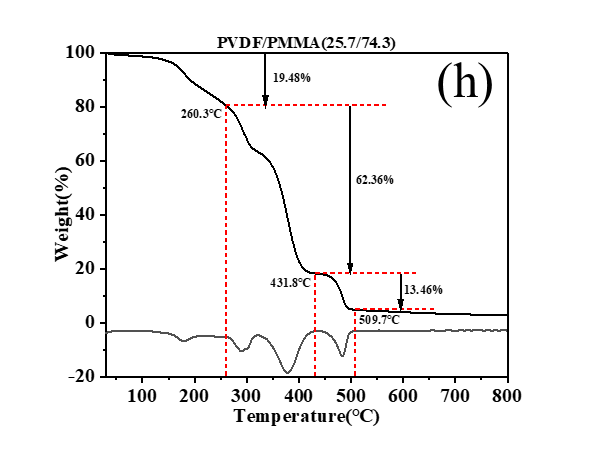** |  |

**Figure S4.** TG and DTG curves of PVDF/PMMA with a MMA content of (a) 0%; (b) 20%; (c) 30%; (d) 40%; (e) 50%; (f) 60%; (g) 70 and (h) 80%

# Small angle X-ray scattering results

PVDF/PMMA blends are estimated by small angle X-ray scattering (SAXS) to obtain the one-dimensional scattering curve. As shown in figure S5, with the increase in temperature, the peak of the one-dimensional scattering curve moves to a lower angle, and the change of peak decreases with the increase of PMMA content. When the PMMA content is greater than 39, there is no obvious peak and the blend exists in an amorphous state.

| **** | **** | **** |
| --- | --- | --- |
| **** | **** | **** |
| **** | **** |  |

**Figure S5.** One dimensional scattering curves of SAXS for the blends with PMMA contents of (a) 0%; (b) 6%; (c) 19.5%; (d) 24.7%; (e) 39%; (f) 56.8%; (g) 62.1 and (h) 74.3%
